# A comparative analysis of longitudinal computed tomography and histopathology for evaluating the potential of mesenchymal stem cells in mitigating radiation-induced pulmonary fibrosis

**DOI:** 10.1038/s41598-017-09021-7

**Published:** 2017-08-22

**Authors:** Jessica R. Perez, Sangkyu Lee, Norma Ybarra, Ola Maria, Monica Serban, Krishinima Jeyaseelan, Li Ming Wang, Jan Seuntjens, Issam El Naqa

**Affiliations:** 1 McGill University, Biomedical Engineering, Montreal, H4A 3J1 Canada; 2 McGill University Health Centre, Medical Physics Unit, Montreal, H4A 3J1 Canada; 3 University of Michigan, Radiation Oncology, Ann Arbor, MI 48103-4943 USA

## Abstract

Radiation-induced pulmonary fibrosis (RIPF) is a debilitating side effect that occurs in up to 30% of thoracic irradiations in breast and lung cancer patients. RIPF remains a major limiting factor to dose escalation and an obstacle to applying more promising new treatments for cancer cure. Limited treatment options are available to mitigate RIPF once it occurs, but recently, mesenchymal stem cells (MSCs) and a drug treatment stimulating endogenous stem cells (GM-CSF) have been investigated for their potential in preventing this disease onset. In a pre-clinical rat model, we contrasted the application of longitudinal computed tomography (CT) imaging and classical histopathology to quantify RIPF and to evaluate the potential of MSCs in mitigating RIPF. Our results on histology demonstrate promises when MSCs are injected endotracheally (but not intravenously). While our CT analysis highlights the potential of GM-CSF treatment. Advantages and limitations of both analytical methods are contrasted in the context of RIPF.

## Introduction

Lung cancer remains the leading cause of cancer death with a very low 15% 5-year survival rate. Radiotherapy (RT) is one of the most commonly used treatments and it is estimated that about 50% of cancer patients will undergo RT at some point during the course of their treatment^1^. Side-effects from RT are a major limiting factor preventing dose escalation for potential better tumor control and overall survival. RT side effects or radiation-induced lung injury occur in about 30% of thoracic irradiation^2^ and can be divided into two phases: an early inflammatory phase named pneumonitis occurring weeks following treatment, and a later fibrotic phase (RIPF) that occurs months to years post-RT. Clinical symptoms include dyspnea, cough, respiratory insufficiency that can seriously impact patients’ quality of life and in extreme cases lead to death. The exact molecular mechanisms behind RIPF remains an area of active research. It involves a cascade of events including direct DNA damage, cell death, the release of inflammatory cytokines, the recruitment of immune cells and the remodeling of the extra-cellular matrix eventually leading to scar formation and lung fibrosis^1, 3^.

Limited treatment options are available for RIPF and involve mostly steroids and oxygen inhalation with marginal success^3^. One of the promising experimental treatment options currently being investigated is the use of Mesenchymal Stem Cells (MSCs). They exhibit immunomodulatory properties, which can help reduce inflammation and further prevent damage leading to fibrosis^4^. MSCs have exhibited great promise in the treatment of pulmonary fibrosis in pre-clinical models^5, 6^. MSCs can be harvested, cultured and then injected directly into the trachea or systemically intra-venously. An alternative and less invasive option, is to stimulate the host’s stem cells so they can be recruited to the site of injury. This can be achieved with the injection of a stem cells-stimulating drug: Granulocyte macrophage colony-stimulating factor (GM-CSF). GM-CSF plays an important role in tissue repair and in the process of pulmonary fibrosis^7^ but its detailed function is still an area of active research. The rationale for using GM-CSF is driven by its ability to mobilize autogenous MSCs in each rat instead of injecting allogenic MSCs from other rats. In addition, Moore *et al*.^7^ demonstrated that the deficiency of GM-CSF enhanced pulmonary fibrosis as a result of impaired production of the potent antifibrotic eicosanoid, Prostaglandin E2. The effect of GM-CSF on mobilization of stem cells and reduction of lung fibrosis has been previously established. In this study, we did not aim to re-evaluate these treatment options at a mechanistic level but rather to contrast their efficacy with serial CT imaging and histopathological analyses as a necessary prerequisite for clinical translation.

Current common approaches to assess and monitor RIPF could be divided into: (1) medical imaging: most commonly computed tomography (CT); and/or (2) histopathology, which remains the gold standard.

Medical imaging can be used to visualize and monitor the onset of RIPF. Radiological evidence of RIPF is part of diagnosis alongside clinical presentation of symptoms^8^. CT is readily available in the clinic and is a powerful tool to image RIPF. CT imaging being based on density changes, the lung is an ideal candidate organ. On CT images, air appears black (low density, −1000 Hounsfield units (HU)), bones appear white (high density, 700 to 3000 HU) and soft tissues are in between with a density comparable to water (0 HU). In the case of RIPF, edema, immune cells infiltrates and fibrosis all contribute to increasing the density of the lungs and therefore, it is possible to visualize those changes non-invasively and longitudinally over time with serial CT imaging^9^. CT density changes have been shown to correspond to the dose of radiation in a mouse model, typically exhibiting changes above 10 Gy^10^. Density and morphological changes were observed in a mouse model of RIPF with CT imaging and interestingly, those changes appeared prior to the onset of clinical symptoms^11^. Rather than qualitatively assessing average density variations or scoring morphological differences, the quantification of RIPF on CT images was proposed by using the density distribution or histogram of the lung in order to get a more reproducible measure independent of the observers^12^. The idea of using CT density changes in monitoring RIPF (and correlating these with radiation dose) is not new^13, 14^ and has been successful and implemented routinely in the clinic. With the advent of new imaging technologies the quality of CT images obtained has increased dramatically and therefore allowed for the detection of more subtle changes, even in small animals. CT density changes over time were previously compared to histological findings in a rat model but did not lead to conclusive correlation with the average lung density most probably because of the focal development of fibrosis in different lung regions^15^. Recently, Reddy *et al*.^6^ demonstrated anti-fibrotic efficacy of MSCs in comparison to pirfenidone in a bleomycin-induced pulmonary fibrosis model using high resolution CT.

Histopathology allows the detection of RIPF at the cellular level with standard evaluation of tissue architecture on hematoxylin & eosin (H&E) slides by a trained pathologist. Specifically, by staining the tissue with fibrosis targeted dye: Masson’s trichrome that makes collagen, the main component of fibrosis, appear blue. Masson’s trichrome staining can be evaluated by visual inspection with a fibrosis scoring scale such as the Ashcroft scale or a modified version of it ref. 16, or quantified using an automated software based on color, determining the amount of blue pixels. Dong *et al*.^5^ used histopathology techniques to demonstrate that anti-fibrotic effects of MSCs on irradiated lungs could be related to host secretions of hepatocyte growth factor (HGF) and prostaglandin E2 (PGE2).

In this work, we assess the potential of different MSCs treatment routes in mitigating RIPF using both CT and histopathology and contrast their findings, advantages and limitations in each case. A rat model of RIPF was established in a clinical setting including CT simulation, treatment planning and radiation delivery on a clinical linear accelerator. Following irradiation, the rats were treated for RIPF with MSCs or drug stimulation and monitored longitudinally with serial CT imaging. Following standard convention, after 24 weeks of follow up, stable RIPF was observed and quantified with histopathology and compared to longitudinal CT images.

## Results

### Onset of RIPF over time on serial CT images

Female Sprague-Dawley rats (Charles River Laboratories, QC, CA) were divided into 5 groups (25 total, n = 5 per group): Non-irradiated control (Control), Irradiated (RT), Irradiated and treated with GM-CSF drug (RT + Drug), Irradiated and treated with MSCs injected intravenously (RT + MSC-IV), and Irradiated and treated with MSCs injected endotracheally (RT + MSC-ET). We followed the rats with serial CT imaging every 2 weeks for 24 weeks post-irradiation. The time line of 24 weeks was chosen based on extensive pilot studies following the onset of fibrosis in our RIPF rat model. It is also consistent with previous literature as in Dong *et al*.^5^ in which the study was terminated after 24 weeks, when fibrosis became stable but animal conditions worsened.

**Figure 1:**
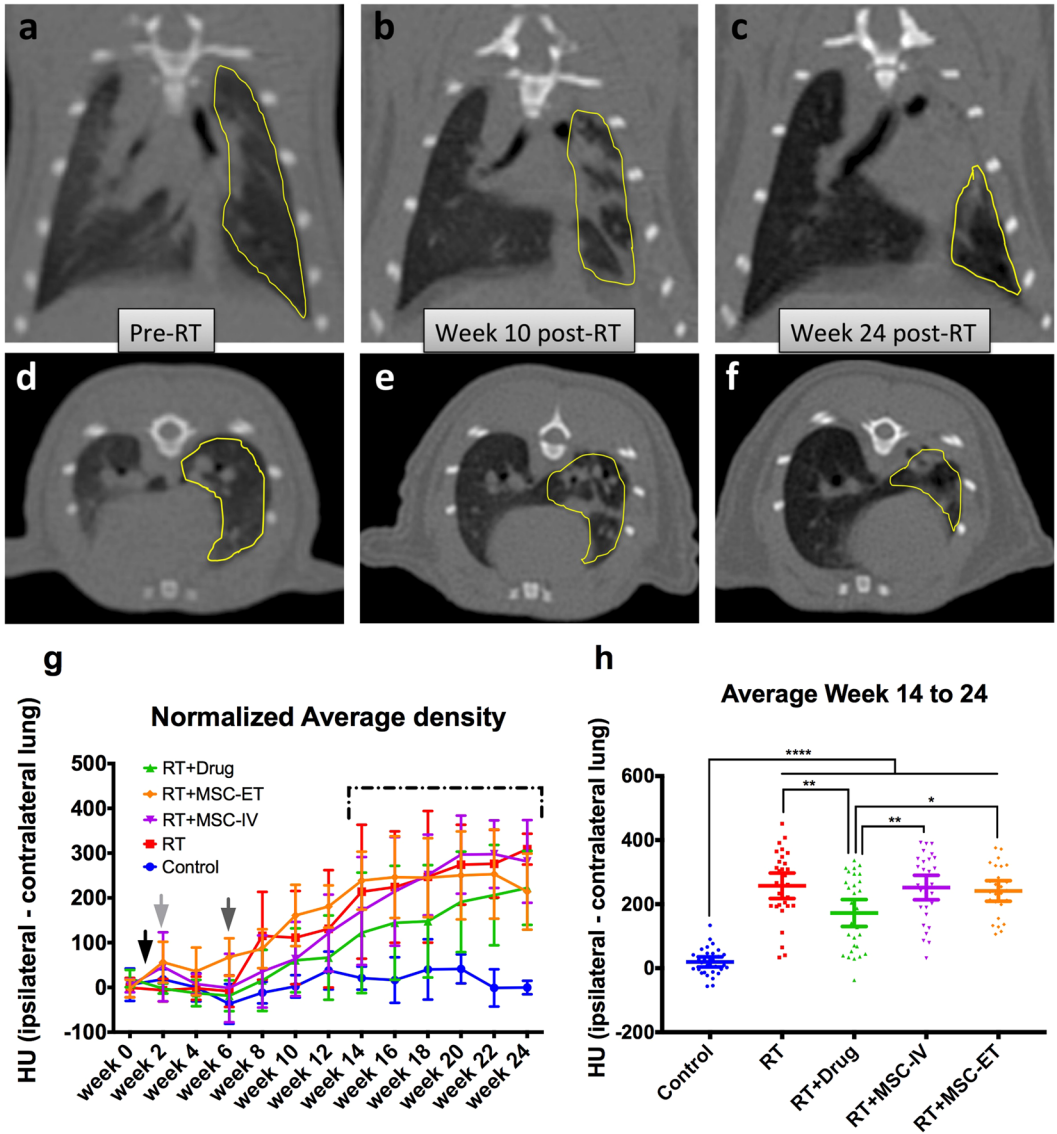
CT images of a representative rat over time. Irradiated lung contour highlighted in yellow. (**a**) Frontal view before RT. (**b**) Frontal view 10 weeks following RT. (**c**) Frontal view 24 weeks after RT. (**d**) Transverse view prior to RT. (**e**) Transverse view 10 weeks following RT. **f**) Transverse view 24 weeks post-RT. (**g**) Normalized average CT density over time for every groups. The Control group lung density remains stable whereas the RT groups show an increased density. Black arrow: RT delivery and start of Drug or MSC injection treatments. Light gray arrow: end of Drug treatment. Dark gray arrow: end of MSC treatments. Dashed line: Averaging period shown in h. (**h**) Average lung density averaged over week 14 to 24 for each group. Bars represent the mean and 95% CI. There is a statistical difference between Control and all RT groups and between RT + Drug and the other RT groups.

Figure 1 shows CT images of a representative rat with irradiated lung. We begin to observe changes in lung appearance visually at 10 weeks post RT (Fig. 1b and e). Lung injury and fibrosis being denser than air, appears brighter on CT images compared to normally aerated lungs. Moreover, we note that the irradiated lung volume shrinks and gets denser over time prior to onset of RIPF. Interestingly, we also observe a shift in the heart and mediastinum towards the injured lung (Fig. 1c and f).

We quantified the density of the irradiated lung (ipsilateral) by taking the average density of the lung region-of-interest (ROI) and normalizing it to the unirradiated lung (contralateral). We observe an increase in density over time in all radiation groups whereas the sham irradiation control group remains stable (Fig. 1g). The RT + Drug group shows a consistent trend of lower densities than the other RT groups (Fig. 1g, green curve). In order to capture differences in density between groups over time, we computed the average of the last 6 time points of follow-up between week 14 and 24 post-RT (Fig. 1h). All RT groups exhibit a statistically significant increase in lung density compared to Control (*p* < 0.0001). The RT + Drug group shows a significantly lower density than the other RT groups (RT + Drug vs RT: *p* = 0.0045; RT + Drug vs RT + MSC-IV: *p* = 0.0094; RT + Drug vs RT + MSC-ET: *p* = 0.0472) (Fig. 1h).

### Fibrosis quantification with CT histogram analysis

**Figure 2:**
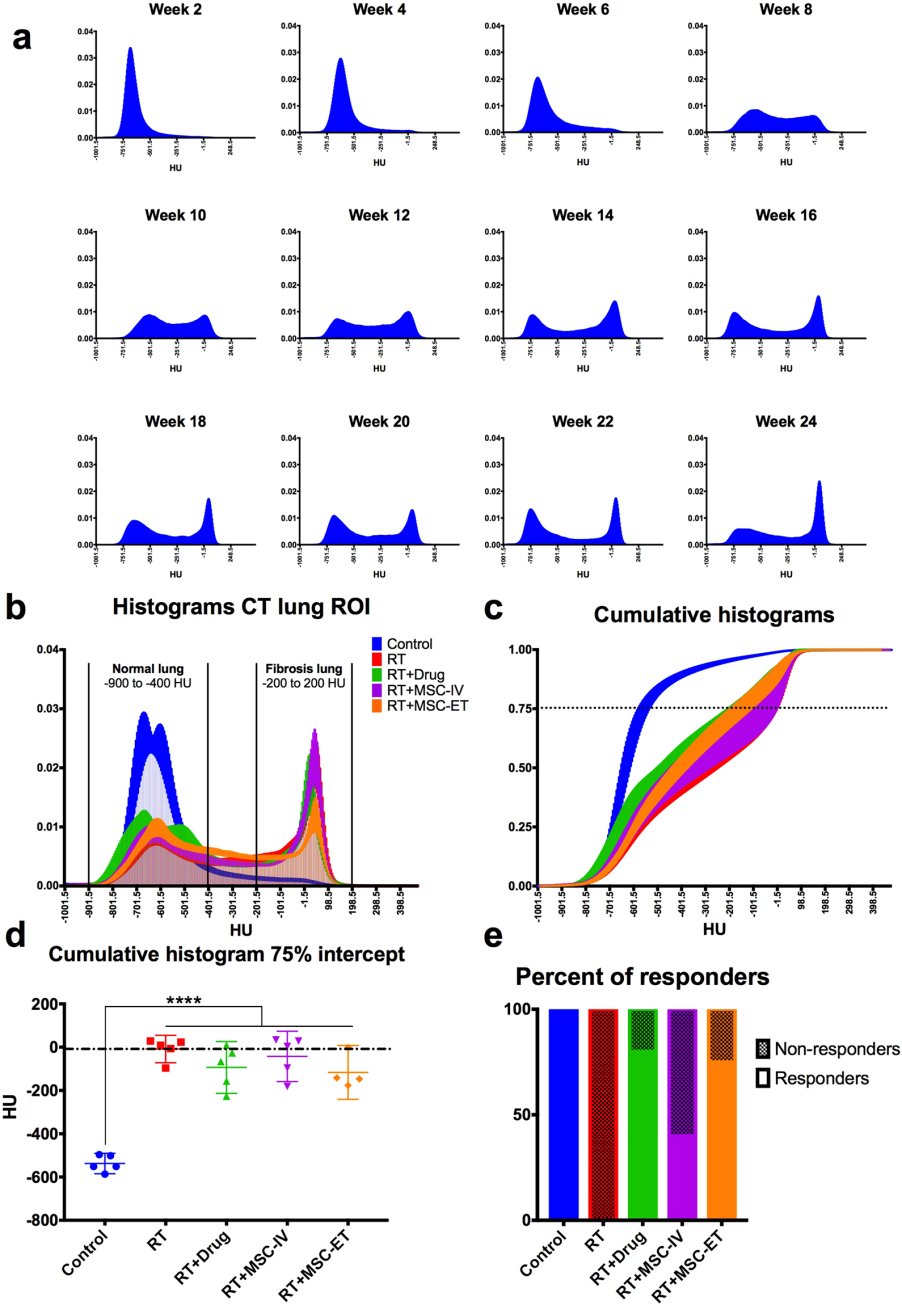
(**a**) CT histograms of lung ROI from a representative irradiated rat over time from week 2 to 24 post-RT. Histograms represents the portion of voxels within the lung ROI at a certain density (HU). (**b**) CT histograms for all groups at week 24 post-RT. (**c**) Cumulative histograms at week 24 post-RT. Dotted line at the 75% intercept line to maximize separation between groups. (**d**) HU value of the 75% intercept from the cumulative histogram for each group. Bar represents the mean with 95% CI and each point is a rat. There is a significant difference between the Control and all RT groups. Dashed line was set at the mean of the RT group. (**e**) Percent of rats exhibiting a response to treatment. Rats falling under the mean of the RT groups (dashed line from (**d)**) are considered “responders” (closer to Controls) and rats above the RT mean are considered “non-responders”.

For each CT image (0.37 mm in plane and 0.4 mm axial resolution), we extracted a histogram of the lung ROI. In the irradiated groups the shape of the histogram varies over time. Figure 2a shows the changes in histogram over time for a representative irradiated lung ROI.

Based on the appearance of the histogram peaks, we qualitatively established two sections of the histograms: (1) the normal lung, ranging from −900 to −400 HU and (2) the fibrotic lung, ranging from −200 to 200 HU (Fig. 2b). In CT, −1000 HU represents air and −500 HU lungs, so the normal lung section exhibits aerated regions of the lung, whereas 0 HU represents water and tissues which indicates poorly aerated lung regions consistent with fibrosis. As shown in Fig. 2a, the fibrotic lung region appears at week 8 and keeps increasing as the number of voxels in the normal lung region decreases. We observe changes in lung appearance visually on CT images at week 10 (Fig. 1b and e). But, when examining quantitatively the shape of the histogram over time, we observe that change takes place earlier at 8 weeks post-RT.

The difference in histograms between the different groups was quantified at week 24 post-RT. Figure 2b shows the combined histograms of all groups. We observe that the control group’s histogram peaks in the normal lung section with small tail in the fibrotic section. In comparison, all irradiated groups show a fibrosis peak with a majority of voxels accumulating in the fibrosis region and a much smaller peak in the normal lung section.

We can also visualize the histograms’ differences by plotting the cumulative histograms for each group. It clearly appears that the Control group reaches unity much sooner than the RT groups (Fig. 2c). In order to quantify the differences between groups, we chose to look at the 75% intercept line (Fig. 2c, dotted line), where the spread among groups is maximal^12^. Figure 2d shows the 75% intercept from the cumulative histogram for all groups at week 24. There is a significant difference between Control and all RT groups (*p* < 0.0001). Despite the limited number of rats per group, we observe that some animals from the treated groups show a response to MSCs treatment as their lung appear less dense than the lungs of the RT group (closer to the Control). Figure 2e shows the percent of “responders” falling under the mean of the RT group (under the dashed line in Fig. 2d. We show that 80% of rats in the RT + Drug group, 40% of the RT + MSC-IV group and 75% of the RT + MSC-ET group exhibit some response to stem cell treatment (Fig. 2e).

### Fibrosis quantification with histopathology

In order to better interpret the CT results, we compared them to the histopathology gold standard. After 24 weeks of follow-up, the lungs were harvested, divided in upper, middle and lower lobes, fixed, sectioned and stained with Masson’s trichrome for fibrosis.

**Figure 3:**
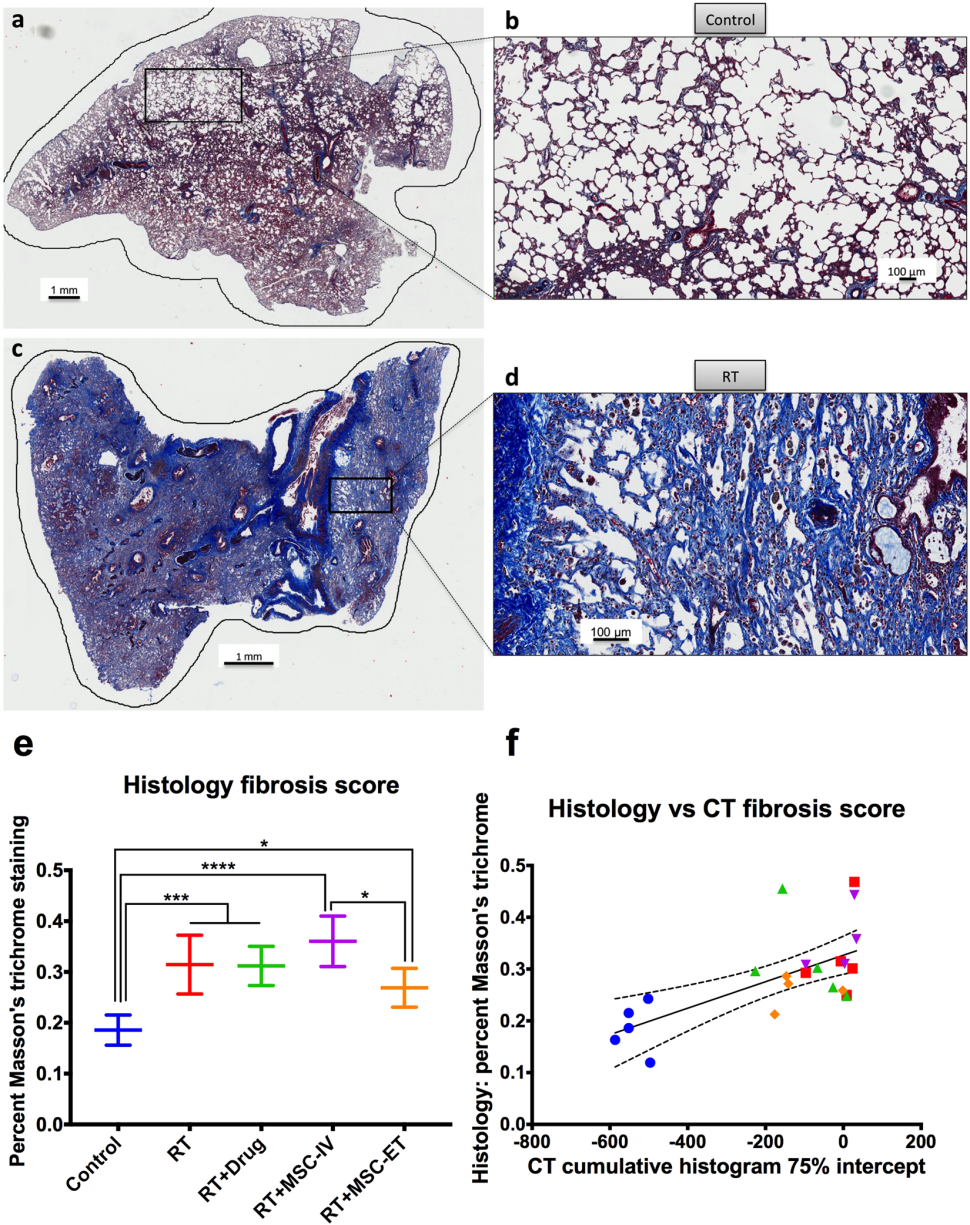
Histopathology of representative lung sections stained for fibrosis with Masson’s trichrome. (**a**) Control whole slide. (**b**) Control zoomed in. (**c**) RT whole slide. (**d**) RT zoomed in. Fibrosis appears in blue. (**e**) Histopathology fibrosis score quantified with percent of blue pixels for each group. There is a statistical difference between Control and all RT groups and between RT + MSC-IV and RT + MSC-ET. (**f**) Plot of histopathology vs CT fibrosis score. Each rat is one point and the line represents the linear regression fit.

Figure 3a–d shows Masson’s trichrome stained lung slides of Control (Fig. 3a and b) and RT (Fig. 3c and d). Whole lung sections from three locations in the lung (upper, middle and lower lobes) were quantified for the percent positivity (the quotient of all blue pixels over all pixels which registers a color expressed as a percentage). Histological data shows an increase of fibrosis in RT (more blue) compared to Control (Fig. 3a and c). Figure 3e shows a statistical difference between Control and all RT groups (Control vs RT and RT + Drug: *p* = 0.0002, Control vs RT + MSC-IV: *p* < 0.0001, Control vs RT + MSC-ET: *P* = 0.0334) and also between RT + MSC-IV and RT + MSC-ET (*p* = 0.0350).

### CT and histopathology comparison

Histology can provide only a snap shot of time. Therefore, we attempted to compare the corresponding CT results from week 24 to the histopathology data. Figure 3f shows fibrosis quantification from CT and histopathology for each rat in a scatter plot. We observe a linear response between increased CT HU and increased percent positivity in histopathology (*R* ^2^ = 0.4123). There is a significant correlation between CT and histopathology scoring (*p* = 0.0008) with Spearman rank correlation *r* = 0.6494.

## Discussion

In this work, we used serial CT imaging to longitudinally monitor the onset of RIPF in a rat model, compared results to histopathology and assessed the potential of different MSCs treatment routes in mitigating RIPF.

It is recognized that the translation to human population is a concern, as we are presenting results from a RIPF rat model. These differences across species as well as between different breeds of the same species have been documented and considered by our group. Some animal models are more radio-sensitive or -resistant than others and that may have an influence on the results of such studies. Translation to the human population is a serious concern for every study done using animal models. However, clinical studies for testing the potential of MSC are limited since the efficacy and safety of using these cells is still controversial, and it is premature to test new risky therapies in humans. Therefore, using such animal models as surrogates to establish a method and monitor a disease state are of great value. In our case, we performed all steps of the experiments under the most possible clinically-like conditions: clinical radiation therapy equipment (clinical Linac and clinical CT) to have a relevant RIPF rat model so the same image analysis techniques could be easily translated into patients.

Pulmonary fibrosis is characterized by the excess deposition of extra-cellular matrix with collagen accumulation as its main component^17^. Therefore, histopathological techniques that specifically stain for collagen deposition (Massons’s trichrome) are considered a reliable measure of pulmonary fibrosis and remain the gold standard in assessing the extent of fibrosis.

We were able to detect changes in CT images as early as 8 weeks post-RT with serial CT histogram analysis. Fibrosis development evolves further on, up to 24 weeks following RT. At this point we were able to directly compare CT information with histopathology fibrosis staining. We observed increased fibrosis in all irradiated groups for both CT and histopathology. In that sense, CT imaging can be a good surrogate to histopathology to monitor RIPF non-invasively and longitudinally.

Our results suggest that treatment with MSCs-ET and endogenous stem cells stimulation with GM-CSF shows promise in the reduction of RIPF. We observe a consistently lower lung density in the RT + Drug group on CT images, from the onset of RIPF, up to the end of our follow up at 24 weeks post-RT. Moreover, histopathology shows less fibrosis in the RT + MSC-ET group. We did not observe an effect when MSCs were injected IV.

The study presents a detailed comparison of different BM-MSCs methods for radiation-induced pulmonary fibrosis and longitudinal evaluation of these methods using serial CT imaging with respect to the gold standard of histopathology at the end of 24 weeks. TGF-*β*1 is considered a key modulator in the onset and progression of fibrosis. The relationship between MSCs and TGF-*β*1 has been previously reported^18–20^. In investigations of co-culture analysis by our group^21^ and by others in the literature^17^ a mechanistic understanding of gene expression was attempted. These types of further mechanistic investigations are, however, beyond the scope of the present study and would be the subject of future research focused on our best candidates for BM-MSCs (ET-MSC and GMCSF).

There are a few caveats in our study. First, we are limited by the number of rats per group (n = 5) since we rely on observing subtle differences and there is a high variability between subjects within any given treatment group. The number of animals per group (n = 5) is standard to obtain sufficient statistical power. We observed similar results in previous pilot studies with n = 3 per group. In accordance with the animal care guidelines, we attempted to use as few animals as possible to still obtain quantifiable results. The effect of current MSCs dosage on mitigating RIPF may be modest and the damage sustained by the lung following a 18 Gy RT might be too high to reverse it. We performed multiple pilot studies with lower and higher radiation doses (16, 18, 20 Gy) to establish the RIPF rat model protocol. Higher doses and full thorax irradiations has led to poor survivability of the rats. Since we delivered RT to one lung only (hemithorax), the other lung was used as an internal control and was able to compensate for the damaged lung. Therefore, survivability is greatly improved and none of the animals died from RT at a dose of 18 Gy to the right lung. We also monitored weights and breathing rates and did not observe significant differences between the RT groups and the controls.

The observation period of 24 weeks was based on our previous pilot studies and similar timelines have been used by others too as in Dong *et al*.^5^. It is noted that longer follow ups could be used but the benefits of longer periods is unclear.

For collagen staining we used Masson’s trichrome, other alternatives are available such as Sirius red or hydroxyproline assays but it is unclear whether this would make a difference in the observed results^22^.

Another uncertainty is associated with the fact that we rely on the MSCs homing or getting recruited to the site of injury. MSCs have a short lifespan, so we are assuming that they survive long enough to have a beneficial effect. Variations in the delivery schemes could also impact the effect of MSCs and more research is still required to determine the optimal treatment dosage.

Fibrosis reduction after MSCs injections was not significant, and this protocol needs further investigation to adjust the MSCs dosage in order to reduce fibrosis significantly. In the current study, we focused on evaluating the therapeutic potential of MSCs when administered via 3 different routes. In future studies, comparing our findings to a control group using fibroblast cells would be also of interest in order to further benchmark the potential of MSCs.

If the acute (inflammatory) phase is controlled it will not proceed to a chronic (fibrotic) phase. Therefore, we decided to provide continuous supply of MSCs once a week for 6 weeks to control the acute inflammatory phase. We followed a previous protocol applied to head and neck irradiated mice^23^ with some modifications established during pilot studies on our RIPF rat model. Perhaps, this treatment dosage was insufficient to minimize or slow down the onset of fibrosis. Indeed, we did not observe a significant delayed onset of fibrosis in the MSCs group compared to irradiation alone on CT images.

Reddy *et al*.^6^ demonstrated the potential of human MSCs transplantation in mitigating pulmonary fibrosis at its early stage in mice and showed consistent results between histopathology and CT, although CT scoring was qualitative. A dedicated small animal scanner was used, however the resolution is comparable to our study. In this scenario, MSCs were administered systemically (IV), but in our case we did not observe any benefit on RIPF in the RT + MSC-IV group. The timeline of the studies is quite different with bleomycin injury leading to much faster onset of fibrosis compared to RT and could potentially explain the differences we see in outcome. Bleomycin injury models do not recapitulate the clinical outcome we are focusing on with RIPF and even though both methods lead to pulmonary fibrosis one should remain cautious as to the exact mechanisms behind the different origins of lung injury. Another critical difference is the timing of MSCs injection, which was performed every week for 6 weeks in our study but within days after bleomycin injury, that can lead to differences in MSCs efficacy. The use of a different animal model (mice vs rats) is very critical when comparing studies as the biological response to lung injury can vary greatly between different breeds of the same species. Dong *et al*.^5^ investigated RIPF in a rat model and demonstrated human MSCs reduced inflammatory factors and histopathological appearance of fibrosis, although histological fibrosis scoring was qualitative. MSCs administration was systemic (IV) and immediately following RT. RT damage in Dong’s study was assessed with histopathology at week 4 post-RT by sacrificing a group of animals at that time point. In our study, we observed RT damage at 8 weeks following RT with CT imaging and not histopathology. We only have histopathological results from week 24 post-RT. In addition, 4 weeks is too early for fibrosis onset at this time, the inflammatory process is still taking place and incomplete. Fibrosis consolidation is ongoing and will take longer to settle following our imaging analysis. In their study the rats received a dose of 15 Gy, which is lower than in our study (18 Gy) and that probably lead to less severe lung damage.

We were able to correlate increased CT and histopathological scores in irradiated groups compared to control. However, the correlation between CT and histopathology results disappears when comparing the different irradiated treatment groups. Both methods have advantages, but also suffer from limitations (Table 1). One such limitation is that we are limited in the resolution of the CT compared to a histopathology slide especially since we are using a clinical CT as opposed to a dedicated small animal scanner. As we used a clinical linear accelerator, we needed a clinical CT simulation in order to plan the treatment for each individual rat and establish feasibility for clinical translation. For its ease for clinical translation, we therefore used the same clinical CT scanner for the follow up period. With an optimized small animal imaging protocol we were able to reach an in-plane resolution of 0.37 mm, which is adequate to resolve fibrotic details within the lung and extract lung densities. The spatial localization of fibrosis is also tricky to correlate between a 3D image volume and a histopathology slide due to the fixation process and orientation of the tissue for sectioning. For this reason, we performed the analysis on the entire lung ROI on the CT and for histopathology we averaged the values of three different lung regions: upper, middle and lower lobes. Another issue to keep in mind is that we used Masson’s trichrome staining that specifically highlights collagen deposition in the tissue, whereas we can only look at increased density on CT images. The biological basis of an increase in lung density is multifaceted and can include aspects such as the accumulation of liquid (edema) or cell infiltrates and of course includes extra-cellular matrix deposition and fibrosis. CT being an anatomical imaging method, only reveals downstream anatomical changes that are a consequence of earlier molecular events. Having a molecular target for fibrosis imaging may allow us to detect these changes earlier. Future research in the development of targeted molecular imaging probes for RIPF will certainly bridge the gap between CT and histopathology.

**Table 1:** Advantages and limitations of CT and Histopathology.

CT		Histopathology	
Advantages	Limitations	Advantages	Limitations
Non-invasive (longitudinal studies)	Anatomical/Macroscopic	Cellular resolution	Invasive/*ex vivo* (sample required)
3D volume	Relies solely on density (not fibrosis specific)	Masson’s trichrome staining is fibrosis specific	Fixation and sectioning affect tissue
Clinically translatable		Tissue architecture	One slice of tissue
		Clinically translatable (biopsies)	Staining procedure

The work presented here is a multidisciplinary effort combining the establishment of a RIPF rat model with MSCs mitigation and monitoring of the disease state with clinically relevant CT imaging compared to the histopathological gold standard. This study contributes to expanding current knowledge of MSC-mediated therapies, including key aspects of cell injection time lines or preferred route of administration, in addition to evaluation methods. More work is indeed needed for stem cells therapy to translate into the clinic and we believe that non-invasive methods based on imaging will play a pivotal role in determining the best ways to achieve this goal.

In conclusion, we were able to monitor RIPF in a pre-clinical setting with CT imaging and histopathology, compared and contrasted both methods for their potential in the assessment of RIPF mitigating MSCs therapies. More work is required to optimize the use of MSCs in RIPF and imaging will be a valuable tool to bring such new therapies to the clinic.

## Methods

### RIPF rat model

All experiments were approved by the Animal Care Committee at the Research Institute of the McGill University Health Centre and in accordance with the ethical guidelines of the Canadian Council on Animal Care.

A rat model of RIPF was established as previously described^24^. Briefly, Sprague Dawley female rats were anesthetized with isofluorane and placed in prone position on an Styrofoam bed. The rats were imaged on a CT-simulator (see section CT imaging for details). Both lungs, the heart and spinal cord were contoured on the CT images. An AP-PA (anterior and posterior parallel opposed fields) radiotherapy plan delivering 18 Gy to the right lung was designed (Eclipse V 11.0) and delivered on a Novalis Tx linear accelerator (Varian Medical Systems, Palo Alto, California, USA) with prior positioning of each rat using cone beam CT.

### MSCs isolation, culture and injection

MSCs were isolated from the bone marrow of male sprague Dawley rats as previously described^25^. Briefly, the femur of the rats were isolated and the bone marrow was flushed out and filtered. The resulting cells were placed in culture in Mesenchymal Stem Cell Growth Medium (MSCGM, Lonza, Switzerland) supplemented with antibiotic-antimycotic (Invitrogen, Thermo Fisher Scientific, USA), that was changed the next day and twice a week following extraction. MSCs were selected for plastic adherence and passaged when reaching 80% confluency. MSCs used for injection did not exceed passage 5. Therefore, MSCs from passage 2 to passage 4 maximum were used for injection. When passaged too many times, changes in size, and potential therapeutic effectiveness of MSCs can affect the results.

MSCs injections were performed the day of irradiation and once a week for 6 weeks following RT. Dosage of MSC injection was obtained from previous extensive pilot studies. The rationale is, if MSCs are provided to irradiated rats for multiple times, this would increase the therapeutic potential of MSCs on lungs compared to controls. We wanted to make sure that MSCs are supplied continuously so that they do not get cleared quickly from the system before having an effect on the irradiated lungs while still not causing harm (such as pulmonary embolism). Cultured MSCs were detached with trypsin-EDTA centrifuged and resuspended in saline for injections. The cells were injected via different administration routes, either endotracheal (ET) (200000 cells) or intravascular (IV) (1 million cells).

Rat GM-CSF drug (Cedarlane, Canada) was administered following irradiation and for the next 7 days via intraperitoneal injection with a dose of 10 *μg*/*Kg*.

### CT imaging

CT imaging was performed for treatment planning prior to irradiation and every two weeks for 24 weeks post-irradiation. CT imaging was performed on a Philips Brilliance Big Bore computed tomography (CT) simulation scanner (Philips Medical Systems, Bothell, WA, USA) using an optimized rat imaging protocol: 120 kVp X-ray tube voltage, 175 mA tube current, 0.37 mm in-plane resolution, 0.4 mm axial resolution.

### Lung CT image analysis

#### Definition of lung region-of-interest (ROI)

Lung was originally contoured for treatment planning on the baseline CT by CT number thresholding (−1000, −300 HU) and the trachea was excluded. First, a 3-dimensional lung ROI on a baseline CT image was created by isotropically eroding (kernel size: 1 mm) the volume within the lung contour drawn for treatment planning. The erosion was performed to minimize the amount of necessary manual correction on the ROI due to registration error. Then, deformable registration was performed between a post-radiation CT images and the corresponding baseline image. The resulting deformation vector field was applied to the baseline lung ROI to create a corresponding lung ROI for the follow-up CT. The image processing procedures were conducted under the MevisLab platform version 2.3.1 (MeVis Medical Solution, Bremen, Germany).

#### Characterization of CT density changes after irradiation

To characterize global anatomical changes in lung, we computed the average lung density and we obtained histogram of CT density in Hounsfield Units (HU) of the voxels within the follow-up lung ROI.

#### Histogram analysis

Lung density values were extracted for each rat ROI at each time point and we generated histograms. We built a cumulative histogram at week 24 and quantified the 75% intercept for each rat.

### Lung tissue histopathology and fibrosis staining

After 24 weeks the rats were euthanized and the lungs were harvested, divided into upper, middle and lower lobes, fixed in formaldehyde and sectioned, transversally, for histopathology. Lung sections were then stained for fibrosis (collagen) with Masson’s trichrome.

### Histopathology staining quantification

Stained lung sections were scanned using a whole slide scanning technique (Aperio™ Leica Biosystems, USA) at 20X magnification. Fibrosis (collagen) regions appear blue with Masson’s trichrome staining, therefore, we quantified the number of blue pixels. To avoid subjectivity in the quantification of histopathology, we used an automatic thresholding method using ImageScope (Leica Biosystems, USA). Each whole-slide image was contoured and large blood vessels and bronchioles were manually removed from the analysis. Then, the color blue was isolated by adjusting the hue values and thresholded to obtain the number of blue pixels. The values represented here are percentages of positivity: number of positive blue pixels over the total number of pixels (representing tissue regions and excluding blank spaces).

### Statistical analysis

Differences between groups were assessed with One-Way ANOVA and correction for multiple comparisons. Correlation between CT and histopathology was performed using Spearman rank correlation.

Significance of p value is represented as follows: *p* > 0.05: ns, *p* ≤ 0.05: *p ≤ 0.01: **p ≤ 0.001: *** and ****p ≤ 0.0001.
